# Parents' Perceptions About Future Digital Parental Support—A Phenomenographic Interview Study

**DOI:** 10.3389/fdgth.2021.729697

**Published:** 2021-10-27

**Authors:** Caroline Bäckström, Sandi Chamoun, Shazima Tejani, Viveca Larsson

**Affiliations:** ^1^School of Health Sciences, University of Skövde, Skövde, Sweden; ^2^Research Group Family Centered Health (FamCeH), University of Skövde, Skövde, Sweden; ^3^Region Jönköping County, Högland Hospital of Eksjö, Maternity Ward, Eksjö, Sweden; ^4^Norra Älvsborg Hospital, Trollhattan, Sweden

**Keywords:** digital health literacy, professional support, pregnancy, childbirth, labor, parenting

## Abstract

**Background:** Parents use digital sources (such as the internet or online forums and applications) during pregnancy and after childbirth to receive informative support. Research shows that there is further need for innovation development in digital parental support despite informative support available in digital form.

**Purpose:** To explore parents' perceptions of future digital parental support concerning pregnancy and the first 18 months of parenthood.

**Method:** A phenomenographic interview study with an inductive approach including 15 semi-structured interviews was conducted.

**Results:** The analysis process resulted in three descriptive categories: Opportunities for virtual and in-person meetings, Individualized digital parental support, and Professional knowledge and trustworthiness concerning future digital parental support.

**Conclusion:** The results broaden the knowledge about how future digital parental support can be designed to facilitate the functional, interactive, and critical digital health literacy of new and would-be parents. To succeed, healthcare organizations should allow healthcare professionals to assume an active role in developing digital parental support, both as health educators (i.e., providing parents with knowledge) and facilitators (i.e., facilitating parents' use of digital parental support). However, parents perceived that future digital parental support should complement standard care instead of replacing in-person meetings with healthcare professionals.

## Introduction

Contemporary society is experiencing a rapid development of digital technology (1), and todays' parents have access to a large amount of different digital sources (2). However, the information provided through these digital sources does not always meet the information *needs* of expecting mothers (3) which often relate to their social or emotional concerns (4). Research shows that there are two kinds of information *experiences* that parents who seek digital information about pregnancy, labor, childbirth, and parenting encounter. On the one hand, expecting mothers use online parenting forums to experience a facilitated feeling of calmness (5) and reduced depression and anxiety (6) about labor issues (7). Meanwhile, expecting fathers also seek information about pregnancy-related topics on the Internet (8) to improve their understanding of fetal development and fragility (9). Furthermore, expecting parents value the opportunity to expand their social network and have their questions answered by social connections online (10). On the other hand, research shows that parents could experience the information online as overwhelming as they include much irrelevant information (11, 12) instead of “local” or “informal” information relevant to the specific parent (3). This problem requires expecting mothers to filter the information (13). They may have difficulties relating to it, which may lead to emotional stress (14) and feelings of lack of social support (3, 14). In addition, previous research has shown that parents prefer information controlled by healthcare professionals (3, 11, 12) because they consider such information credible, reliable, understandable, and applicable in their life situation (15).

However, the information parents receive online is sometimes not in line with recommendations from healthcare professionals (16), or local hospital policies (3). This observation is worth noticing because expecting mothers tend to turn to online sources when feeling abandoned by professionals (14) or filter the information they received from midwives (17). Besides, their search for online information impacts their decision-making regarding pregnancy-related issues (18). Research from different intervention studies has shown that parents benefit from using online programs developed by healthcare professionals: mindfulness programs reduced expecting mothers' depressive or anxious symptoms (7, 19), and a cognitive behavioral therapy program for expecting mothers reduced anxiety, distress, and depression (20). Meanwhile, research also shows that when online programs are compared with other forms of programs, there are no significant differences in the emotional health concerns of expecting mothers (21). Mothers who fear childbirth are more satisfied with face-to-face counseling than they are with internet-based Cognitive Behavioral Therapy (22). Subsequently, research results differ in reporting the consequences of parents using digital sources for information seeking and social support during the parental transition.

Becoming a parent is a vulnerable time in a person's life (23). Both professional support (i.e., support from healthcare professionals such as midwives) and social support [i.e., support from social contacts such as family, friends, and significant others; (24)] are known to facilitate a smooth parental transition (25). Expecting mothers who have lost proximity to their family due to relocation may feel isolated during pregnancy and after childbirth due to a lack of social support (26). Becoming a parent in a new country can inhibit new and different demands. Furthermore, specific needs are linked to being an immigrant parent because these parents are in a more or less unknown context (27, 28) during the sensitive parental transition (23). In addition, expecting mothers seek information online concerning their specific sociocultural needs for information and support (14). Donelle et al. (29) described the parental transition as a period filled with intense emotions, especially when reaching information for individuals and families, discussing normality, social communication, and trustworthiness of digital health information. They suggest that digital sources are applicable for areas of parents' empowerment together with self-management of antenatal and postpartum care. Furthermore, they identified a need for future research to understand parents' digital health literacy skills and health outcomes and bridge inequities in healthcare services and use of online health information. Another aspect worth noticing in care for parents is the need to enhance parents' digital health literacy skills (consisting of several literacies), both regarding health information seeking from digital sources and knowledge to tackle health issues (29). One example highlights the empowering processes for parenthood, concerning not only information but also development of skills, to gain, understand and use information on parents' and their children's health (30). Three different dimensions have been described for health literacy: *functional health literacy* (i.e., individual reading, writing, and handling of everyday life), *interactive health literacy* (i.e., cognitive and social skills and support for participation and communications in everyday life), and *critical health literacy* [i.e., abilities for analysis, critique, and control of information, social, political, and organizational agency; (31)]. In a review of the health literacy concept, the importance to include social relationships in the concept is identified together with a processual approach life long learning (32). Recent work updates health literacy with digital communication and media use (33), and interaction in digital discussion forums has been described to influence the maternal health literacy (34). The information needs and experiences that parents identify, viewed through a process of life long learning, will here be discussed as a possibility of a future parental digital health literacy.

The needs of expecting and new parents in accessing information through digital sources and developing a parental digital health literacy are complex, which differ based on their circumstances. To fully meet parents' needs for digital parental support in the future, it is crucial to explore this area of research further. Such exploration should include a variety of parents representing both expecting and new parents and first- and multiple parents experiencing the sensitive parental transition. These parents should also represent various cultures and languages to broaden the knowledge about how future digital parental support could be designed to meet culturally diverse population of parents experiencing parental transition within the same country. Hence, this study aimed to explore parents' perceptions of future digital parental support concerning pregnancy and the first 18 months of parenthood. In this study, *digital parental support* is used as an umbrella term for different digital sources, such as the internet or online forums and applications. When referring to specific sources, these are specified in the text.

## Materials and Methods

### Design

The current study is part of a larger research project, *Digital Parental Support*, which includes explorative and mixed methods studies, employing both inductive and deductive approaches. The larger research project aims to explore two different serious games for parents, while the current study forms part of a pre-study that explores parents' perceptions of future digital parental support concerning pregnancy and the first 18 months of parenthood. The present study used explorative design, qualitative method, inductive approach, and phenomenographic analysis (35). Qualitative methods aim to broaden an understanding of a specific phenomenon by conducting data collection within peoples' natural settings. An inductive approach allows research to be grounded in the participants' narratives (36). Phenomenography derives originally from pedagogical research and has become more commonly used within nursing research in recent years. Researchers explore peoples' various perceptions or different ways of making sense of and understanding phenomena within phenomenography. It is the underlying structure of variance in the perceptions that are discovered, rather than the actual phenomenon itself, as in many other qualitative research methods (37). In the current study, the phenomenon explored was parents' perceptions of future digital parental support concerning pregnancy and the first 18 months of parenthood.

### Setting and Participants

The study setting was two regions in southwestern Sweden, representing a population of ~15,000 inhabitants. The researchers conducted the recruitment between autumn 2019 and winter 2020 using convenience sampling. The recruited participants were also part of another research project, *Reinforced Parenting—Extended Home Visits* (38). Child healthcare (CHC) nurses asked parents who met the inclusion criteria of their interest to participate. The ideal participants for the study were parents who lived in the setting and expected or had had a child for the last 18 months. Eleven mothers and four fathers participated, representing an age range between 23 and 57 years old. Eleven participants were Swedish-speaking. The other participants were Arabic speaking, which means that these interviews were conducted in Arabic with the help of a professional translator. Meanwhile, one interview was conducted in English. Nine of the participants had a high school education, 11 expected or had a child younger than 18 months, and four had another child between 1 and 7 years old (Table 1). Thus, the participants represented various expecting, new, and more experienced parents who knew about the phenomenon to be explored. Therefore, the number of participants was considered satisfactory. The study included parents who participated individually and those who participated as a couple.

**Table 1:** Participant characteristics.

**Women**	**Men**	**Age**	**Swedish** **speaking**	**Non-swedish** **speaking**	**High school** **education**	**Expected or had** **a child younger** **than 18 months**	**Expecting first-time** **parent or parent** **with a child** **younger than** **18 months**	**Parent with at** **least one child** **older than 1 year**
11	4	23–57 years	8	7	9	11	11	4

### Data Collection

The responsible managers at public CHC centers provided written approval for conducting the recruitment process within the regions. The researchers prepared a semi-structured interview guide consisting of open-ended and follow-up questions, as such guides are commonly used in phenomenographic traditions (37). The researchers initially tested the interview guide in three pilot interviews to explore informant interpretation of the interview questions. The three participants in the pilot interviews did not participate in the *Reinforced Parenting—Extended Home Visits* project. The results from the pilot interviews showed that the questions were easy to understand and respond to. All interviewees met the inclusion criteria, and their descriptions met the aim of the study. Therefore, the pilot interviews were included in the data analysis. Examples of interview questions are: *How do you think information and support can be provided digitally to expectant and new parents? How do you perceive that future digital support for parents could be designed concerning pregnancy and the baby's first 18 months of life?* Follow-up questions were used to broaden the participants' narratives, such as: *Could you explain further? Three* authors (BC, CS, and TS) were responsive to the 15 interviews conducted between November 2019 and January 2020. The interviews were held in the participants' homes or at the CHC centers. They lasted between 19 and 61 min. Three of them were held with a parental couple together. The others were held with the participants individually. One interview was done in English, three were conducted in Arabic with a professional translator, and the rest were conducted in Swedish. The interviews were recorded digitally and transcribed verbatim in Swedish (14 interviews) or English (one interview). In all, the transcript corresponded to 108 A4 pages of text with single-line spacing.

### Data Analysis

For data analysis, the phenomenographic method was used, and six of the seven analysis steps (35) were followed: (1) *Familiarization*, all transcripts were read repeatedly by three of the authors (BC, CS, and TS) to obtain an overall understanding of the data; (2) *Compilation*, narratives that corresponded to the aim of the study were marked and gathered into statements; (3) *Condensation*, to obtain a representative description of the parents' perceptions, the different statements were concentrated; (4) *Grouping*, statements that were familiar with each other were grouped together, in total 11 groups derived; (5) *Comparison*, similarities, and differences between the groups of statements were identified to find distinct borders between the groups, which resulted in reduction into four groups in total; (6) *Naming*, perceptions, and emerging descriptive categories were discussed among all authors, and named to highlight their essence with an adequate level of abstraction.

### Ethical Considerations

The responsible managers approved the current study at public CHC centers and the Regional Ethical Review Board in Gothenburg (Dnr 2019:03906). The participants received written and verbal information in their chosen language and the opportunity to ask questions before they gave their consent to participate. A professional translator described the study and responded to questions for the parents who did not understand the Swedish language. Participation was voluntary and could be canceled by the participants without giving a reason for why. Participants' identities were handled with confidentiality, which meant that only the researchers had access to their identities, and the transcripts were unidentified before data analysis. Therefore, the results are presented with participant quotes that do not reveal participants' identities.

## Results

The analysis resulted in three descriptive categories with perceptions, as presented in Table 2.

**Table 2:** Overview descriptive categories and perceptions.

**Descriptive categories**	**Perceptions**
Opportunities for both virtual and in-person meetings	Virtual meetings with healthcare professionals Virtual meetings with other parents In-person meetings with healthcare professionals cannot be replaced by digital technology only
Individualized digital parental support	Conditions for individualized information Adapted to parents' various abilities to handle digital technology Available in multiple languages for equality Information conveyed with variety may facilitate parents' interest and understanding
Professional knowledge and trustworthiness to future digital parental support	Conveyed by healthcare professionals to facilitate trustworthiness Consistent with information provided in real meetings with healthcare professionals

### Opportunities for Both Virtual and In-person Meetings

The parents perceived that future digital parental support should provide opportunities for both virtual (using digital technology) and in-person (physical meetings in real life) meetings with healthcare professionals (such as midwives) and other parents. The parents perceived opportunities for in-person meetings with healthcare professionals as valuable. They emphasized that such meetings cannot be replaced by digital technology in the future.

#### Virtual Meetings With Healthcare Professionals

The parents saw future digital parental support as a shortcut to contact healthcare professionals, such as midwives, in urgent or acute situations. So, for example, if a pregnant woman should end up in a critical or life-threatening situation, digital parental support could provide opportunities for expecting parents to quickly get into direct contact with a midwife who could then support them. According to the parents, such support could be provided through chat forums where parents could communicate with healthcare professionals. One participant said: “*It should not only be information but also opportunities for a direct contact… Maybe something happens to the pregnant woman; then she can directly get in contact with the midwife and see her, it would be easier*” (Participant #10).

Furthermore, the participants believed that future digital parental support could reduce waiting times for parents. They meant that parents would not have to sit and wait in a hospital or healthcare center if they could receive professional support digitally in the comfort of their home instead. For the participants, expecting parents could experience increased freedom through digital parental support because they would receive support while doing household tasks. One participant said: “*The most important thing is that with this* [digital parental support that allows virtual meetings with healthcare professionals] *you can avoid these long waiting times at clinics, emergency rooms, and hospitals… then you will be more free when waiting at home… you do not sit and wait for a ward* [at the hospital]” (Participant #14).

#### Virtual Meetings With Other Parents

The parents expressed that they wanted future digital parental support to allow parents to have virtual meetings with other parents. In such meetings, parents could discuss practical issues regarding pregnancy and the baby's first years. Furthermore, they could share experiences and converse about the various challenges of parenthood and how such challenges could be handled. Hence, the parents could learn from and support each other. In that way, new social ties could be developed. Besides, the parents perceived it as positive if such virtual communications between different parents were monitored by healthcare professionals who could provide concrete answers and comment on the chat between the parents. One participant shared:

*The world overall is digital… you can do anything in the digital world… develop an app for expectant mothers… you can be placed in a group with other mothers who are expecting babies during the same period… organize language cafés, it can start digitally … create a language café and sit there for two hours and lead a conversation… people would still talk to each other digitally both after and before… they* [healthcare professionals] *should create a website, maybe a channel… where mothers can talk about their experiences as a parent… Listening to people's stories is helpful* (Participant #3).

Future digital parental support could, according to the parents, create opportunities for different kinds of parents to meet virtually—parents with different cultural backgrounds and languages, for example. Such meetings could positively affect the information exchange related to issues on parenthood within different cultures. According to parents' perceptions, this could inhibit future parents' feelings of loneliness and facilitate feelings of social cohesion. A participant said: “*If you have pregnant mothers… You can organize a meeting or a digital language café about pregnancy and everything… when you move to a new country you are completely alone… It can be very difficult… It can be a mixture of both. I think everyone can have something to bring into the conversation. So, Swedes may have information that foreigners do not have and Swedes may be interested in knowing how it goes in other countries*…” (Participant #3).

#### In-person Meetings With Healthcare Professionals Cannot Be Replaced by Digital Technology Only

The parents perceived that future digital parental support should complement standard care instead of replacing in-person meetings with healthcare professionals. According to the parents, virtual meetings via digital technology such as telephone, video meetings, or other digital communication sources could not replace in-person meetings between them and healthcare professionals because in-person meetings allow emotions and empathy to a more considerable extent. The parents appreciated in-person meetings (i.e., face-to-face meetings). They described such as more secure and trustworthy. They believe that in-person meetings facilitated increased opportunities to receive information based on their individual needs compared with virtual meetings. A parent said: “*I still want to meet a person, I want to talk face-to-face… that is more important than sitting over a phone or the web or a webcam or whatever… I think it facilitates a feeling of security to get to know or meet a person… although it is good to get the information digitally as well… but, it does not replace the real contact with the healthcare staff…*” (Participant #12).

### Individualized Digital Parental Support

This descriptive category describes how parents' perceptions of future digital parental support should be adapted to meet parents' individual needs and circumstances. Such digital parental support could, according to the parents, facilitate prospective parents' sense of having their needs of information satisfied.

#### Conditions for Individualized Information

The parents perceived that future digital parental support should promote conditions for prospective parents to receive individualized (i.e., person-centered) information. They pointed out that the existing information on the internet is not adapted for all parents. Hence, it is general and usually not based on the unique needs of a specific parent's situation. In addition, the parents described the search for information on the internet as sometimes broad and sometimes limited. Search results include large amounts of various answers that do not always fit the specific parent's situation. In addition, future digital parental support could consist of particular web links to other websites of interest or alignment of registers and search engines on the internet.

*The information should be based on the specific person* [person-centered]*, if I have pain somewhere, there are various factors that affects* [the situation]… (Participant #5).

Future digital parental support should, according to the parents, promote individualized information at the right time and the right pace for the user. Hence, knowledge is based on the specific phase of life the individual parent is currently experiencing (i.e., pregnancy or the child's first 18 months). Furthermore, future digital parental support should allow parents to obtain specific information aimed at the parent, i.e., the pregnant woman, expecting father, new mother or father, single, or homosexual parents. The parents perceived that such individualized information could lead to future parents experiencing less anxiety and frustration when consuming information digitally.

*The information for the partner should be different for female partners and male partners… also, it may be a good thing that you would be able to choose… if you choose to have information for single mothers, then it* [the information] *does not show the partner's* [situation or role] (Participant #3).

#### Adapted to Parents' Various Abilities to Handle Digital Technology

The parents perceived that future digital parental support should be user-friendly and attractive for parents with high and low digital consumption and parents with various digital abilities for information searching. They meant that different parents possess a variety of experiences and levels of education, and, therefore, they have a varied skill set in digital information searching. Hence, future digital parental support should allow more or less advanced ways to search for information. Parents who have higher abilities should have the possibility to search for information in a more advanced way. The parents pointed out that the ability to handle digital information searches will probably affect what information a parent absorbs. Therefore, they perceived it as necessary that future parents support their ability to sort out adequate information on relevant websites to obtain relevant, reliable information. An example given was on issues regarding children with physical or mental problems. When searching for information related to this topic, parents need to find relevant and trustworthy information.

*Not all people are able to find what they are looking for online, some do not have previous experiences with Internet search* [on parental issues]…* not everyone has the right level of education to find the right type of information*… (Participant #15).

#### Available in Various Languages for Equality

Non-Swedish-speaking parents perceived that future digital parental support should be presented in Swedish and in English and other languages. For example, they perceived that translation programs could be integrated into various digital sources, where the user can choose which language the information should be translated into. However, a translated text can lead to misinterpretations, according to the parents. Parents appreciated when various films were presented by other parents telling their experiences and stories as new parents. They described that such movies are already available on different Swedish websites, including the official national healthcare website, but only in the Swedish language. Non-Swedish-speaking parents perceived that information and films could be provided in other languages for equal healthcare treatment and equality with future digital parental support. According to the parents, there should be no linguistic difference in the amount of information provided for parents. Information on today's Swedish websites differs in the amount of text between the Swedish and English versions. Commonly, the information in Swedish includes detailed and informative text, while the English version is shorter. The parents perceived that all future parents should have the same right and opportunities to receive support in performing their parental role, regardless of their language skills.

*So, really ensure equality, everything that is available to Swedish parents should be available to foreign parents as well* (Participant #3).

Future digital parental support should also bring the opportunity for parents to receive tips and advice on which sources of information to use. The non-Swedish-speaking parents perceived that today's information is limited and not as accessible for them. These parents expressed a need for comprehensive practical details. They wanted more detailed information about things they should bring with them to the labor and postnatal ward. They wanted information about how long they could stay in the hospital. In addition, parents wanted information about their partner's and midwife's role during childbirth. They wanted information about Swedish hospital routines, baby care, baby's winter clothes (especially foreign parents who previously lived in countries with a warm climate), and suggestions for strollers and baby car seats they should procure.

*There is not much in English on Swedish websites, there is not much available for non-Swedish speakers… it is not so helpful… the information I wanted to know, I did not find online… I have no idea how long you stay in the hospital, I do not know what to bring to the hospital. I do not know what role the partner plays during the birth and the days that follow* (Participant #3).

#### Information Conveyed With Variety May Facilitate Parents' Interest and Understanding

The parents perceived that future digital parental support should offer information that appeals to parents and their interests. For example, they pointed out that information about the fetus is both of great importance and excitement. Furthermore, future digital parental support should offer easily accessible information that is comprehensive and free of charge. One parent said: “*I think information provided digitally should be comprehensive, very comprehensive. I mean, it should be accessible*” (Participant #10). They agree that such information should be conveyed visually in images or films. They said that images could be pedagogical and supportive. For example, suppose their child has rashes on their skin. In that case, future parents can compare these with the images conveyed in the digital parental support, which gives the parents a specific idea of what is normal and what is abnormal. According to the parents, in some cases, images are more easily comprehended than text. Images could help them decide if and when contact with healthcare should be initiated. The parents also think that films included in future digital parental support could be of great value, especially in emergencies when parents must act quickly and correctly. Examples of such situations could be administering cardiopulmonary resuscitation in children or addressing breathing difficulties among children.

A parent said:

*Maybe some kind of heart and lung rescue on children… if the child gets something in the throat, and then maybe examples of symptoms such as this with breathing difficulties… I searched for that at some point, it was very difficult to find examples of that, how it was shown in reality* (Participant #4).

The parents perceived that today's digital technology could allow entertaining functions in future digital parental support. For example, designers and developers could employ a serious game to make information more engaging. As parents seek and obtain the information they need, they can have fun as well. What's more, couples could use such moments together to deepen their bond. In the words of a parent, “*With today's* [digital] *technology, there are many other ways, especially if you want to capture the parents' interests… Maybe you can make a* [serious or interactive] *game of any kind*…” (Participant #1).

### Professional Knowledge and Trustworthiness Concerning Future Digital Parental Support

This descriptive category includes the parents' perceptions regarding the value of reliable and trustworthy information provided by healthcare professionals. It also contains the importance of evidence-based and reassuring information in which future parents can relate to their current situation.

#### Conveyed by Healthcare Professionals to Facilitate Trustworthiness

The parents considered that healthcare professionals should convey the information provided in future digital parental support. The parents perceived that the current information on the Internet could lead to feelings of concern and doubts. They perceived that healthcare professionals, such as midwives or medical doctors, are up-to-date and possess high-level competence regarding pregnancy, childbirth, pediatric (baby) care, and parenthood-related issues. Therefore, the information provided by such professionals becomes trustworthy, according to the parents. Hence, future digital parental support should function as a communication link between healthcare professionals and prospective parents, with opportunities to interact and ask questions directly to a professional and thus receive reliable answers immediately.

A participant expressed this through the following statement:

*Maybe it would have been good… if there was someone… some midwife who sat in such a group* [of parents]…* that it* [the group conversation] *is controlled by a midwife in that case… if there are people who are not educated, then they can express and believe various things, and that can be very wrong* (Participant #5).

According to the parents' perceptions, the information provided in future digital parental support should be reviewed by professionals and based on reliable scientific facts. People without relevant professional education should not be allowed to mediate such information. The parents believed that information solely based on other parents' experiences could lead to the reader experiencing uncertainty or anxiety: “*I would prefer facts from professionals… it is reliable facts, yes, more than personal experiences. Experiences can differ very much and it is not always that someone else's experiences will be like mine… you may then doubt yourself… it can lead to more worries*” (Participant #2).

#### Consistent With Information Provided in In-person Meetings With Healthcare Professionals

The parents perceived future digital parental support as a possible reassuring source of information. For this to occur, the information provided should be consistent with the information provided by healthcare professionals during in-person meetings. Then, future parents would be able to compare, value, and confirm information received. In addition, future digital parental support should facilitate prospective parents' feelings of trustworthiness and security because they will see it as an opportunity to receive reassuring, professionally conveyed, and updated information. In the words of a participant: “*You get about the same message if you ask about things. So it still feels well that you can trust things that are online… there is a little more confirmation*…” (Participant #4).

## Discussion

The results of this study showed that parents' perceptions of future digital parental support concerning pregnancy and the first 18 months of parenthood suggest a need for opportunities for both in-person and virtual meetings and individual support from professionals who demonstrate expertise and trustworthiness. This section will discuss parents' perceptions, present needs, and experiences related to parental digital health literacy in the making, discussed through different aspects.

Couples have always experienced emotional challenges as they make the transition into parenthood, but today's would-be parents are facing a new challenge: a colossal amount of digital sources providing them with information (2, 3, 29). The interviewees in this study described health information as part of functional health literacy and as an element of individualization in future digital parental support, adjusted to parents' specific role, language, and context, unlike the web-based general information of today. This aligns with health literacy as a personal asset (39) and discusses health literacy related to the socio-economic context (33). As may be interpreted in the current interviewees' perceptions on the ability to tackle digital sources and information (included in functional health literacy) may also refer to sociocultural needs (14). Therefore, the results of this study suggest that future digital parental support should include an element of individualization, or personalization, to better meet the unique needs of individual parents. Then, the future digital parental support could be proactive in guiding parents to obtain relevant information.

Interviewees in the current study discuss interactive health literacy in terms of learning with entertainment and the suggestions of using interactive (i.e., serious) games in future digital parental support. In addition, the interviewees discuss empowering participatory processes (interactive health literacy) in the context of meetings between parents and professionals, including virtual and face-to-face interactions. Interviewees propose that future digital parental support could strengthen and empower prospective parents' (interactive health literacies) in social skills and participation in everyday activities (31). Interviewees in the current study also described future digital parental support as a possible facilitator for parental engagement and interactivity when discussing personalization and adjustment to social backgrounds, such as suggestions of virtual language cafés and similar spaces. Parental engagement and interactivity are also considered in other research to expand parents' social networks, online connections, and their ability to discuss issues (10). This is in line with the interviewees' perception that future digital parental support that includes virtual meetings between parents could initiate discussions on parental issues in different social contexts or cultures and even prevent feelings of isolation and loneliness in a new country. Previous research about the need for social support during relocation (26) and specific needs of refugee parents in a new context (27, 28) support these facts. In addition, interviewees discussed interactivity concerning healthcare professionals. They suggested that future digital parental support should include opportunities for immediate online contact with healthcare professionals in cases when parents are in urgent need. They indicated that such options in future digital support could reduce waiting times for parents, which they perceived as positive. As one dimension of health literacy, interactivity could thus accentuate a need for synchronic (e.g., parents in need of immediate professional support) and diachronic (e.g., interaction not bound to a particular point in time, like serious games, or online forums) parental participation online.

Interviewees in the current study also expressed a need for future digital parental support to include information about practical issues like healthcare routines, baby care, etc. This present study and previous research show that, in both online and real life, parents do not always face consistent information from peer parents and healthcare professionals (3, 16). In addition, information online could vary in quality (29). To nurture parents' critical health literacy, including their ability to analyze information, healthcare professionals should actively develop digital parental support. They could do this both as health educators (i.e., providing parents with knowledge) and facilitators (i.e., facilitating parents' use of digital parental support). The parents' expressed need for professional expertise and trustworthiness in future digital parental support aligns with the possibilities of using the critical health literacy dimension to tackle life events and situations with more control (31).

Accordingly, the interviewed parents in this study seem to prefer healthcare professionals to control the information included in future digital parental support. However, it remains unsaid whether this need is related to the parents' personal insecurity or current healthcare organizational constraints that do not always allow them access to professional knowledge. However, by identifying their own needs for professional knowledge, the parents in the current study show the ability to analyze their needs concerning current information available through digital sources. This is worth highlighting because expecting mothers tend to turn to online sources when feeling abandoned by professionals (14) or to control the information received from midwives (17). In addition, would-be parents' unmet needs for information negatively affect their sense of being prepared for childbirth and parenting (40, 41). Nevertheless, the interviewees in this study discuss a need for dedicated future digital parental support. For this, healthcare professionals should control the information and, thereby, evaluate the knowledge behind the information. In connection with this, other studies have found that parents are sometimes overwhelmed when faced with irrelevant information online (3, 11, 12). Through education and facilitation, the professionals may enhance parents' analytic and critical-thinking skills, i.e., assist them in becoming critically health literate. Indeed, putting healthcare professionals in control of evaluating information may be one of the most significant areas of development in parental digital health literacy.

Donelle et al. (29) discuss tailored postnatal health literacy parental courses with health literacy content to build health skills and knowledge among parents. This could be an embedded content in various future digital parental support and the aspects identified by the interviewees in this study. Future digital parental support could also cover and encourage empowerment, bridging inequalities in health services, use of online information, and self-management of prenatal and postpartum care, as suggested by Donelle et al. (29). Benefits from professionally developed online programs have been stated elsewhere (7, 19, 20). However, both face-to-face and virtual interactions are needed, according to both the results of the current study and previous research (22).

The results of this study broaden the knowledge of parents' perceptions of future digital parental support and suggest some vital aspects that need to be included in future support for parents. Previously, Robinson et al. (3) have stressed the importance of digital information portals that are user-centered designed to optimize its' benefits for both parents and healthcare professionals. This knowledge could be used by healthcare organizations and professionals developing digital parental support. Besides, the elements could be used by healthcare professionals who care for becoming or new parents. Hence, the results of the current study suggest parents' various perceptions of digital parental support, which is in line with previous research showing that parents' experiences using digital sources to obtain information related to childbirth and parenting vary. However, the perceptions of future digital parental support described by the parents in this study could be affected by the parents' previous information experiences. Hence their different experiences of various types of interactivity and visualization when obtaining information may have impacted on their perceived trustworthiness of the interaction. Nevertheless, with an ambition to promote health for parents and their children in a digital society, we see a need for an ongoing adjustment of parental support to the digital spaces where parents interact and create trust and confidence with each other and the profession, and what it means to be health literate for future parents. Therefore, we suggest that healthcare professionals who care for becoming or new parents create spaces for discussions about both parents' general use of digital sources and the unique use among the individual parent. To initiate such conversations with parents, professionals could use questions like “*How do you obtain information about childbirth and parenting?*” “*How do you experience such information-seeking?*” and “*What do digital sources for information-seeking and social connections mean to you?*” Although the informants of the current study were a limited number of parents living in restricted regions in Sweden, the informants represent a wide variety of parents with different cultural backgrounds, a strength within phenomenographic traditions (37). Therefore, the results might be transferable to parents living in other countries and theoretically transferable to related questions. However, a limitation of this study could be its use of a professional interpreter in some of its interviews. This may have affected the interviewees' abilities to feel relaxed and understood during the interviews or negatively affected the interviewers' ability to understand informants' narratives and ask follow-up questions. On the other hand, the interpreter was professional and experienced in the study setting. Through the interpreter, the study was able to tap participants from various backgrounds, a quality essential to the study.

## Conclusion

This study showed that parents' perceptions of future digital parental support concerning pregnancy and the first 18 months of parenthood included a need for opportunities for both in-person and virtual meetings and individual support representing professional knowledge and trustworthiness. The results broaden the knowledge about how future digital parental support can facilitate becoming and new parents' digital health literacy, both functional, interactive, and critical. Today's parents are using various digital sources to obtain information and connect with and broaden their social networks. However, there is room for improvement of digital parental support, according to current results. Future support should include opportunities for parents to interact with both healthcare professionals and other parents. To succeed, healthcare organizations should allow healthcare professionals to play an active role in developing digital parental support, both as health educators (i.e., providing parents with knowledge) and facilitators (i.e., facilitating parents' use of digital parental support). However, the parents perceived that future digital parental support should complement standard care instead of replacing in-person meetings with healthcare professionals.

## Author's Note

Contemporary society is experiencing a rapid development of digital technology, and todays' parents have access to a large amount of different digital sources. On the one hand, parents use online parenting forums to experience a facilitated feeling of calmness and reduced depression and anxiety. On the other hand, research shows that parents could experience the information online as overwhelming as they include much irrelevant information. To fully meet parents' needs for digital parental support in the future, it is crucial to explore this area of research further. The knowledge conducted in this study suggests that future digital parental support should be designed to facilitate the functional, interactive, and critical digital health literacy of new and would-be parents. Specifically, the results showed that future digital parental support should allow: opportunities for both virtual and real meetings between parents and healthcare professionals; individualized digital parental support based on professional knowledge to facilitate trustworthiness. Healthcare professionals should assume an active role in developing future digital parental support, both as health educators (i.e., providing parents with knowledge) and facilitators (i.e., facilitating parents' use of digital parental support).

## Data Availability Statement

The datasets presented in this article are not readily available because we do not have permission to share data. Requests to access the datasets should be directed to caroline.backstrom@his.se.

## Ethics Statement

The studies involving human participants were reviewed and approved by Ethical Review Board in Gothenburg (Dnr 2019:03906). The patients/participants provided their written informed consent to participate in this study.

## Author Contributions

CB contributed to the study's design, ethical application, conceptualization, data curation, formal analysis, investigation, methodology, validation, visualization, and as well as to writing the article, which is an original draft. SC and ST contributed to the conceptualization, data curation, formal analysis, investigation, methodology, validation, visualization, and approved the final version of the article. VL contributed to the formal analysis, validation, interpretation of data for the work, and writing of the article. All authors contributed to the article and approved the submitted version.

## Funding

This study has been funded by the School of Health Sciences at the University of Skövde, Sweden. As well as the Research Group Family Centered Health (FamCeH), University of Skövde, Sweden.

## Conflict of Interest

The authors declare that the research was conducted in the absence of any commercial or financial relationships that could be construed as a potential conflict of interest.

## Publisher's Note

All claims expressed in this article are solely those of the authors and do not necessarily represent those of their affiliated organizations, or those of the publisher, the editors and the reviewers. Any product that may be evaluated in this article, or claim that may be made by its manufacturer, is not guaranteed or endorsed by the publisher.
