# Corrigendum: Parents' Perceptions About Future Digital Parental Support—A Phenomenographic Interview Study

**DOI:** 10.3389/fdgth.2021.805357

**Published:** 2021-12-08

**Authors:** Caroline Bäckström, Sandi Chamoun, Shazima Tejani, Viveca Larsson

**Affiliations:** ^1^School of Health Sciences, University of Skövde, Skövde, Sweden; ^2^Research Group Family Centered Health (FamCeH), University of Skövde, Skövde, Sweden; ^3^Region Jönköping County, Högland Hospital of Eksjö, Maternity Ward, Eksjö, Sweden; ^4^Norra Älvsborg Hospital, Trollhattan, Sweden

**Keywords:** digital health literacy, professional support, pregnancy, childbirth, labor, parenting

In the original article there was an error in the authors list, and the names had the first name and surnames reversed. The correct author list is as follows: Caroline Bäckström, Sandi Chamoun, Shazima Tejani and Viveca Larsson.

Bäckström Caroline, Chamoun Sandi Tejani Shazima and Larsson Viveca

Caroline B, Sandi C, Shazima T and Viveca L (2021) Parents' Perceptions About Future Digital Parental Support—A Phenomenographic Interview Study. *Front. Digit. Health* 3:729697. doi: 10.3389/fdgth.2021.729697

Due to this error the following sections have been revised.

## Author Contributions

The author contribution statement has been revised from:

BC contributed to the study's design, ethical application, conceptualization, data curation, formal analysis, investigation, methodology, validation, visualization, and as well as to writing the article, which is an original draft. CS and TS contributed to the conceptualization, data curation, formal analysis, investigation, methodology, validation, visualization, and approved the final version of the article. LV contributed to the formal analysis, validation, interpretation of data for the work, and writing of the article. All authors contributed to the article and approved the submitted version.

to:

CB contributed to the study's design, ethical application, conceptualization, data curation, formal analysis, investigation, methodology, validation, visualization, and as well as to writing the article, which is an original draft. SC and ST contributed to the conceptualization, data curation, formal analysis, investigation, methodology, validation, visualization, and approved the final version of the article. VL contributed to the formal analysis, validation, interpretation of data for the work, and writing of the article. All authors contributed to the article and approved the submitted version.

Citation

The citation has been revised from: Caroline B, Sandi C, Shazima T and Viveca L (2021) Parents' Perceptions About Future Digital Parental Support—A Phenomenographic Interview Study. *Front. Digit. Health* 3:729697. doi: 10.3389/fdgth.2021.729697

to

Bäckström C, Chamoun S, Tejani S and Larsson V (2021) Corrigendum: Parents' Perceptions About Future Digital Parental Support—A Phenomenographic Interview Study. *Front. Digit. Health* 3:805357. doi: 10.3389/fdgth.2021.805357

The authors apologize for this error and state that this does not change the scientific conclusions of the article in any way. The original article has been updated.

## Publisher's Note

All claims expressed in this article are solely those of the authors and do not necessarily represent those of their affiliated organizations, or those of the publisher, the editors and the reviewers. Any product that may be evaluated in this article, or claim that may be made by its manufacturer, is not guaranteed or endorsed by the publisher.

